# Outcome indicators used in recovery college effectiveness studies: a scoping review

**DOI:** 10.3389/fpsyt.2026.1969790

**Published:** 2026-10-22

**Authors:** Yosuke Seike, Aya Bakuroda, Maki Tamari

**Affiliations:** Faculty of Social Welfare, University of Kochi, Kochi, Japan

**Keywords:** effectiveness, mental well-being, outcome indicators, outcome measures, personal recovery, recovery college, scoping review

## Abstract

Recovery Colleges (RCs) are educational, recovery-oriented approaches in mental health. Differences in evaluation purposes and settings may lead to diverse outcome indicators, making it important to map their use and supporting measurement evidence. This scoping review aimed to identify the outcome indicators used in RC evaluations and clarify their measured constructs, patterns of use, and reporting of reliability and validity. Methods: The review followed JBI methodology and PRISMA-ScR reporting guidance. PubMed, Web of Science, CINAHL Ultimate, ERIC, and seven databases accessed through ProQuest were searched. English-language journal articles reporting quantitative RC evaluations, including quantitative components of mixed-methods studies, were eligible. Eligible data included questionnaire scores before and after RC participation and health service utilization indicators, such as hospital admissions and service contacts. Three reviewers independently screened titles and abstracts and assessed full texts. Data were extracted collaboratively by two reviewers and independently checked by a third. Measured constructs were classified using inductive content analysis. Results: Of 336 records identified, 149 remained after deduplication and 22 articles were included. Thirty-nine measure and indicator entries were identified across three domains: intra-personal and psychological changes, behavioral and social changes, and system and environment. The Warwick-Edinburgh Mental Wellbeing Scale (WEMWBS), including its short version (SWEMWBS), appeared in 11 articles (50.0%) and the Questionnaire about the Process of Recovery (QPR) in four (18.2%); 28 entries appeared in one article only. Fifteen articles (68.2%) cited previous psychometric information or identifiable validation literature, two (9.1%) explicitly reported within-study reliability estimates, and six (27.3%) reported neither specific psychometric evidence nor an identifiable validation citation. One article was not applicable to scale psychometrics. Conclusion: RC evaluations used diverse measures, while direct assessment of educational processes and outcomes was comparatively limited. This review mapped measurement choices rather than RC effects. The findings can inform co-produced decisions about outcome selection and study design, alongside further evaluation of existing instruments’ suitability for the intended RC populations. Involving learners and other stakeholders in these decisions can help align evaluation with research purposes and RCs’ educational philosophy while ensuring that participants’ individual goals are also assessed.

## 1 Introduction

Recovery Colleges (RCs) have evolved as an educational practice based on adult learning models to support the recovery of individuals experiencing mental health challenges (1, 2). A core feature of RCs is the emphasis on co-production, which integrates the experiential knowledge of individuals with lived experience and the expertise of professionals (2, 3). In addition to co-production, RCs emphasize educational rather than clinical approaches, strengths-based and self-directed learning, equality between learners and educators, social connectedness, and community orientation (2). Notably, not only individuals with mental illness but also family members, supporters, and health and social care professionals can participate as “students.” In recent years, RCs have been positioned not merely as an individual recovery support intervention, but as open learning spaces within the community and a novel model of mental health practice (3, 4). Since the first RC opened in England in 2009, the model has expanded internationally, with RCs now operating across diverse mental health systems and cultural contexts (3, 5). This expansion has occurred alongside growing international recognition of recovery-oriented care in mental health policy and practice, emphasizing opportunities to lead meaningful lives beyond symptom reduction. RCs offer a practical educational approach consistent with this shift, supporting the development of knowledge, skills, and social connections (6).

Research on RCs has steadily accumulated over the past decade. A 2020 literature review demonstrated the breadth of RC research, reporting that various outcomes have been investigated, including personal recovery, well-being, goal achievement, knowledge acquisition, self-management, stigma reduction, and service utilization (3). A subsequent co-created scoping review synthesized approaches used to evaluate RCs and highlighted considerable variation in evaluation designs, methods, and outcomes (7). Furthermore, a 2025 state-of-the-art review synthesized 64 RC evaluative studies published between 2013 and 2024 (4). This review highlighted that while RC research has progressed from implementation studies to outcome evaluations, significant heterogeneity remains in study designs and evaluation methods. It concluded that consolidating evaluation studies and streamlining measurement tools are critical future challenges (4).

Previous reviews have synthesized the development and evaluation of RCs, including their implementation, evaluation approaches, and reported outcomes (3, 4, 7). Building on these broader syntheses, the present review focuses specifically on the quantitative outcome measures and objective indicators used across RC effectiveness studies. Its contribution is to integrate, at the measure level, the constructs assessed, patterns of use, and reporting of reliability and validity. For instance, recent related studies have focused on specific aspects, such as a document analysis of RC course content in England (6) and a scoping review of outcome measures used in RC dementia courses (8). The present review complements this work by providing a structured inventory across RC evaluation contexts, linking what has been measured with how it has been measured and what psychometric evidence has been reported.

This focused synthesis can support the accumulation of knowledge in RC research and help contextualize cross-study comparisons. In contrast to conventional symptom-focused approaches, RCs represent a multifaceted practice embodying values and principles such as well-being, personal recovery, empowerment, learning, co-production, and social participation (2, 3, 9). Given the educational foundations of RCs, evaluation should also consider whether current outcome measurement captures learning-related processes and changes, including knowledge acquisition, self-directed and collaborative learning (4, 9). This educational perspective also directs attention to learner agency, identity development, and the transfer of learning to everyday life, including how learners make choices, develop valued roles, and apply knowledge and skills beyond the classroom (6). Moreover, because co-production is a core principle of RCs, consideration of meaningful outcomes should extend beyond researcher-defined indicators and incorporate the perspectives of people with lived experience and other RC stakeholders (7).

Accordingly, clarifying what is evaluated as an outcome and what scales are used for measurement is a prerequisite for adequately understanding how RC outcomes have been evaluated. In addition, examining whether reliability and validity are reported for the measures used can provide information on the psychometric evidence supporting current measurement practices. However, agreement on which outcomes matter—what to measure—is distinct from selecting and evaluating instruments to assess them—how to measure. The former requires consideration of the priorities of learners and other stakeholders; the latter requires evidence that an instrument’s measurement properties are adequate for the intended population and purpose. Mapping psychometric reporting can identify the evidence described in RC evaluations and gaps requiring further investigation, but it does not itself establish instrument suitability or determine a core outcome set. Together with the constructs assessed and patterns of measure use, this mapping can inform measure selection and study design and, if a core outcome initiative is pursued, provide supporting information for stakeholder deliberation and subsequent instrument evaluation. Therefore, this scoping review aimed to identify the outcome indicators used in RC effectiveness studies and to synthesize their measured constructs, patterns of use, and reporting of reliability and validity. Specifically, the review addressed the following questions (1): What outcomes have been evaluated in RC effectiveness studies? (2) What measures and objective indicators have been used to assess these outcomes and how frequently? and (3) What evidence of reliability and validity has been reported for the measures used?

## 2 Methods

### 2.1 Study design

This scoping review aimed to identify and synthesize outcome measures and objective indicators used in quantitative evaluations of RCs, including the quantitative components of mixed-methods studies. The review was conducted in accordance with the JBI methodology for scoping reviews (10) and reported following the PRISMA extension for Scoping Reviews (PRISMA-ScR) (11). The review mapped the constructs assessed, patterns of measure use, and reporting of reliability and validity. A review protocol was prepared but was not registered and is not publicly available.

A scoping approach was selected to map the range and conceptual coverage of measures across diverse evaluation designs, rather than estimate pooled intervention effects or rank instruments.

### 2.2 Literature search

The initial searches were conducted in PubMed, Web of Science, and CINAHL Ultimate on March 8, 2026. To broaden coverage of education and social science literature, additional searches were conducted in ERIC and through the ProQuest platform on September 8, 2026. The ProQuest search was conducted simultaneously across seven databases: Coronavirus Research Database, Education Research Index, MEDLINE, ProQuest Dissertations & Theses A&I, PTSDpubs, Publicly Available Content Database, and Research Library.

All searches covered publications dated on or before March 8, 2026, with no lower publication-year limit. The additional searches retained this publication cutoff to align with the initial searches.

The search strategies combined terms related to RCs with terms related to outcomes, evaluation, and measurement. Strategies were adapted to the syntax requirements of each database or platform while maintaining equivalent search concepts. The complete search strategies, search dates, publication limits, and numbers of records retrieved are presented in **Supplementary Table S1**. Language and publication-type eligibility criteria were applied during screening.

### 2.3 Eligibility criteria

Studies were eligible if they directly evaluated RCs, including Recovery Education Centers and Discovery Colleges operating within the RC model, and reported quantitative data collected or analyzed to assess participant outcomes, perceived impacts, or the recovery-supporting environment and program provision. Quantitative studies and mixed-methods studies with quantitative evaluation components were eligible. Measures could include standardized instruments, study-specific questionnaires, individualized goal indicators, service-use data, and economic indicators. Cross-sectional evaluations were eligible when the quantitative data were used for these evaluative purposes. Studies conducted solely to develop or validate an instrument, without evaluating RC-related outcomes, were excluded. Qualitative-only studies, reviews, protocols, and descriptive reports without eligible quantitative evaluation data were also excluded.

Eligibility was restricted to original research and service evaluation articles published in English in academic journals and reporting methods and results. The English-language restriction reflected the review team’s language capabilities. Non-English publications, conference abstracts, dissertations, and grey literature, including local service evaluation reports published outside academic journals, were excluded.

### 2.4 Study selection

Search results were managed in Google Sheets. Duplicate records within and across search sources were identified and removed by comparing DOIs; when a DOI was unavailable, titles were compared. Three reviewers (YS, AB, and MT) independently screened all titles and abstracts against the eligibility criteria. All articles retained for full-text assessment were subsequently evaluated independently by each of the three reviewers. Disagreements at either stage were resolved through discussion among all three reviewers, with re-examination of the relevant articles until consensus was reached.

### 2.5 Data extraction

Two reviewers (YS and AB) jointly extracted data using a shared Google Sheets form, checking the source articles together. The form was piloted on seven randomly selected candidate articles before eligibility decisions were finalized. No changes to the form or recording rules were made after piloting. Following completion of data extraction, a third reviewer (MT) independently checked all extracted data against the source articles. Thus, extraction was conducted collaboratively by YS and AB, followed by independent verification by MT, rather than independent duplicate extraction.

The form included authors, publication year, country, title, DOI, relationship to RCs, whether the article reported an evaluation study, study design, participant characteristics and sample sizes, reported outcomes, measurement instrument names, measurement time points, whether measures were standardized or study-specific, and reported information on reliability and validity. Eligibility decisions and the reasons for these decisions were recorded in the same form.

Disagreements concerning data extraction were discussed by all three reviewers (YS, AB, and MT). Each reviewer’s rationale and the relevant passages in the source articles were examined until all three reviewers reached consensus; decisions were not determined by majority vote.

Information on reliability and validity was extracted as reported in the included articles, including the types of properties described and numerical estimates, such as Cronbach’s alpha and intraclass correlation coefficients. Information explicitly pertaining to short or modified versions was recorded for those versions. Cited instrument development and validation studies were not independently retrieved or assessed.

An integrated inventory was created by consolidating repeated occurrences of the same measure across included articles into a single entry. Full and short versions of the same instrument were grouped under one instrument entry. Where an article used only a subscale or a modified version, the specific content used was retained in the extraction notes. Alternate names were consolidated only when they were identified as referring to the same instrument, with version-specific information retained. Standardized instruments, study-specific measures, objective indicators, and cost-related indicators were included in the same inventory, with their types distinguished.

Study-specific questionnaires were consolidated only when they were identified as the same measure. Self-reported service use was distinguished from record-derived utilization. “Standardized” denoted a previously developed instrument, rather than a judgment that its measurement properties were adequate in the included evaluation. Separate entries could represent components of the same questionnaire and were therefore not necessarily independent instruments.

### 2.6 Data analysis

Frequency of use was calculated as the number of included articles reporting each measure. This analysis described patterns of measurement use in RC evaluations and identified commonly used and less frequently reported measures. Frequency was interpreted as an indication of use, rather than evidence of measurement quality or suitability. Grouping full and short versions for frequency analysis did not imply that their psychometric properties were equivalent.

Each article contributed at most one count to each inventory entry. Article-level counts were used rather than counts of participants or independent cohorts. Study characteristics and measurement contexts were summarized descriptively to aid interpretation of differences in measure use.

Outcome measures were categorized using inductive content analysis to identify conceptual patterns across the diverse measures used in RC evaluations (12, 13). This approach was selected because the review concerned the meaning and conceptual focus of measures, including study-specific and objective indicators, for which instrument names alone were insufficient. Inductive content analysis allowed categories to be developed from the extracted descriptions rather than imposed from an existing recovery framework.

Two reviewers (YS and AB) jointly examined the descriptions provided in the included articles, the constructs measured, and the subscales to identify the principal measurement focus of each measure. These conceptual descriptions formed the basis for coding and grouping measures according to similarities and differences. Categories were developed inductively and iteratively merged, split, or renamed as the analysis progressed.

A third reviewer (MT) reviewed the resulting classification. When this review identified differences in interpretation or category assignment, all three reviewers discussed the relevant descriptions, constructs, and subscales until consensus was reached. Measures spanning multiple domains were assigned to the single domain that best represented their principal measurement focus, based on their closest conceptual fit.

The analysis yielded three domains: (1) Intra-personal and psychological changes, (2) Behavioral and social changes, and (3) System and environment. These domains describe the conceptual focus of the measures and do not imply that every included study assessed change over time.

Psychometric reporting was summarized descriptively by measure. The summary distinguished between evaluations conducted within the included study sample and references to previous validation research, based on the descriptions in the included articles. Information not reported in an included article was recorded as “not reported.” This synthesis characterized the reporting of measurement properties rather than formally appraising their quality. Frequency of use, reported psychometric information, and measurement quality were treated as distinct considerations.

For article-level summaries, reporting was classified as previous psychometric information or identifiable validation citations, confirmed within-study reliability, within-study validity information, unclear provenance, no reported psychometric information, or not applicable to scale psychometrics. Statistical validity reporting and non-statistical face/content descriptions were distinguished. Categories could overlap, and percentages used all included articles as the denominator. Coefficients with unclear provenance were not counted as confirmed within-study estimates. A source citation alone was not treated as a validation citation.

Consistent with the mapping purpose of this scoping review, no formal critical appraisal or risk-of-bias assessment of the included studies was conducted. Psychometric reporting was not treated as a formal evaluation of study quality or instrument quality.

## 3 Results

### 3.1 Literature search and study selection

As a result of the literature search, a total of 336 records were identified: 80 from PubMed, 40 from Web of Science, 76 from CINAHL Ultimate, 13 from ERIC, and 127 through the ProQuest platform. After merging these records and removing 187 duplicates, 149 records were screened by title and abstract. Of these, 122 were excluded, leaving 27 reports sought for retrieval. Three reports could not be retrieved. The remaining 24 full-text reports were assessed for eligibility, and two were excluded because they concerned instrument or fidelity development without evaluation of RC-related outcomes. Twenty-two articles were ultimately included in this review (**Figure 1**; **Table 1**).

**Figure 1:**
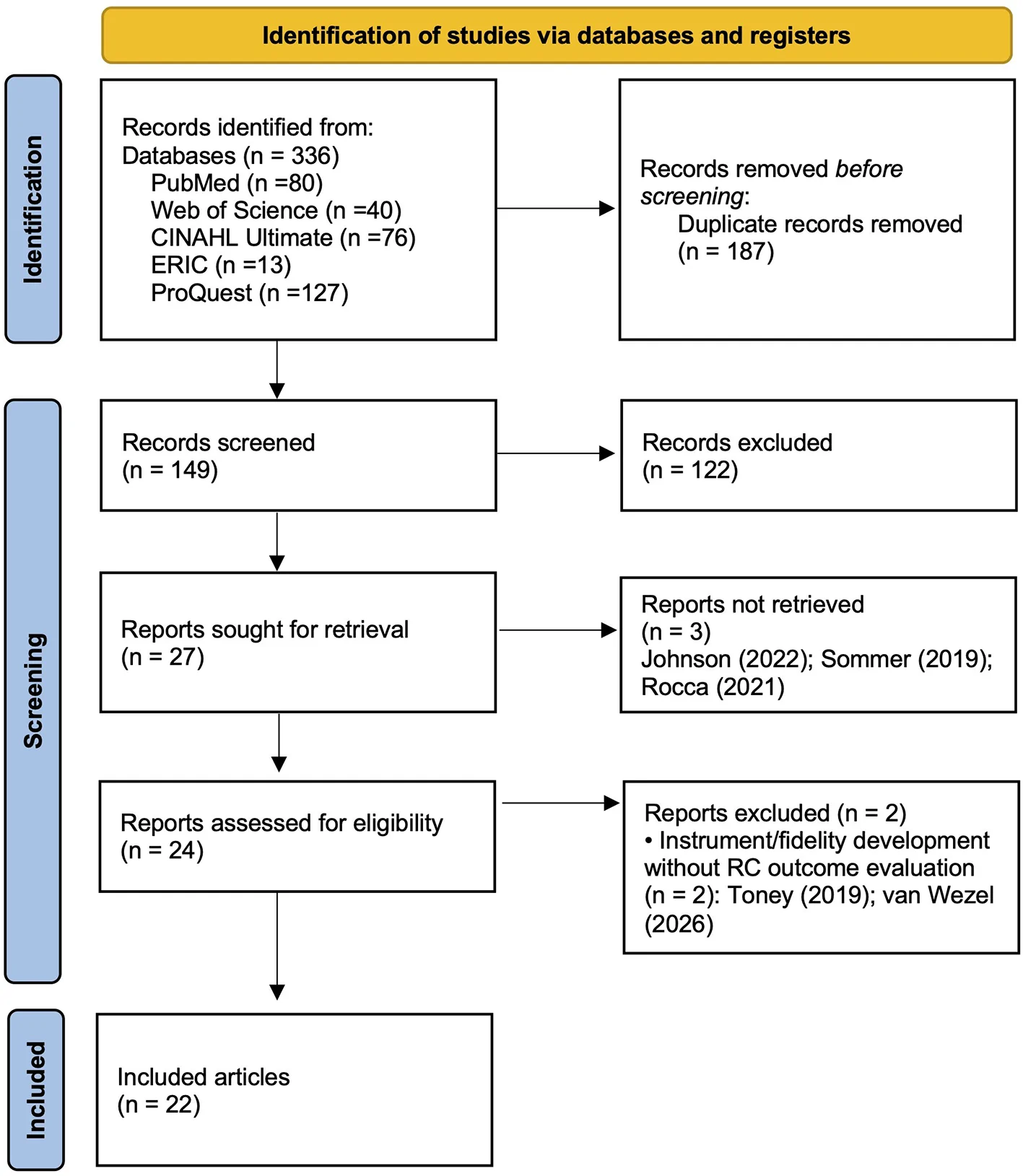
PRISMA 2020 flow diagram of study selection.

**Table 1:** Characteristics of included studies (n = 22).

Author (year), reference	Country	Study design	Target population	Sample size	Measurement time points
Allard et al. (2024) (17)	UK	Pre- and post-intervention study	RC students (service users)	n = 101	Baseline, post-intervention (or 3 months)
Ambord et al. (2025) (28)	Switzerland	Mixed-methods study	RC students	n = 92	Baseline, post-intervention
Arbour et al. (2023) (29)	Canada	Pre- and post-intervention study	RC students	n = 32	Post-course (retrospective baseline)
Bourne et al. (2018) (22)	UK	Retrospective cohort study	RC students	n = 463	18 months pre-registration, 18 months post-registration
Briand et al. (2023) (25)	Canada	One-group pre-post study with repeated measures	Online RC learners, including health/education staff, people with lived experience, students, relatives and other citizens	n = 59 at baseline; 45 at T1; 47 at T2	Before training (T0), within 2 weeks post-training (T1), 3 months post-training (T2)
Briand et al. (2024) (9)	Canada	One-group pretest-posttest quasi-experimental study with repeated measures	RC students	315 consented; 261 at T0; 227 at T1; 205 at T2	Baseline, post-intervention, 3-month follow-up
Burhouse et al. (2015) (18)	UK	Quality-improvement service evaluation with pre–post self-report measures and qualitative focus groups	Pop-up RC students: people with mental health difficulties, family/friends and professionals; optional post-course coaching	n = 50 across three courses; outcome-specific response denominators not reported	Before and after the course; exact interval not consistently reported
Cartier et al. (2026) (21)	UK	Observational mixed-methods service evaluation	Adult RC members living with mental health problems in the community; Feldenkrais and vocal improvisation workshop	n = 8 in the evaluation: 7 completed questionnaires and joined the focus group; 1 joined the focus group only	Questionnaires: pre-workshop, immediately post-workshop and 2-week follow-up; SWEMWBS: pre-workshop and 2 weeks post; focus group: next day
Cronin et al. (2023) (20)	Australia	Cost-benefit analysis	RC students/healthcare system	n = 184	12 months pre-registration, 18 months post-registration
Doroud et al. (2024) (16)	Australia	Mixed-methods evaluation	RC students, supporters, staff, educators and external stakeholders	82 participants overall; 74 DREEM respondents	Cross-sectional
Durbin et al. (2021) (14)	Canada	Longitudinal outcome evaluation	Adults transitioning from homelessness	n = 184	Baseline, 6-month and 12-month follow-up
Ebrahim et al. (2018) (19)	UK	Mixed-methods service evaluation with pre-post quantitative measures	RC students attending courses over three terms in 2016	n = 56 with paired scale data; 89 course-feedback forms	Registration/start of term and end of term or course; mean interval: 51 days (SWEMWBS), 52 days (Empower Flower); maximum: 131 days
Kay et al. (2019) (31)	UK	Service evaluation and data review	RC students/staff	n = 176	Pre- and post-term; 12 months pre- and post-enrolment for service-use data
O’Brien et al. (2024) (30)	Ireland	Multimethod evaluation; cross-sectional quantitative survey	RC students, peer educators, facilitators, coordinators and service providers	n = 94	Initial survey; planned pre–post comparison not completed
Perkins et al. (2017) (15)	UK	Mixed-methods study	NHS staff	n = 94	Post-attendance retrospective survey
Rapisarda et al. (2022) (26)	Canada	Pre-experimental study	RC students (online RC)	n = 107	Baseline, post-intervention, 3-month follow-up
Rapisarda et al. (2025) (27)	Canada	Longitudinal study (cluster analysis)	RC students (online RC)	n = 353	Baseline, 1-month post, 3-4-month follow-up
Řezníková et al. (2025) (34)	Slovakia	Mixed-methods study	RC students	n = 44	Pre-course, post-course
Stevens et al. (2018) (23)	UK	Mixed-methods study	RC students (arts courses)	n = 37 students and 11 trainers	Pre- and post-course questionnaires; 3-, 6- and 9-month qualitative follow-up
Sutton et al. (2019) (33)	UK	Quantitative evaluation	RC students	n = 108	Baseline, 9-month follow-up
Wilson et al. (2019) (24)	UK	Mixed-methods study with a one-group pre-post quantitative strand	RC students with mental health difficulties	n = 101 at baseline; paired data: 11 at 3 months; 25 (SWEMWBS) and 19 (SIS) at 6 months; 17 focus-group participants	Baseline, 3- and 6-month follow-up; three focus groups
Yoeli et al. (2022) (32)	UK	Mixed-methods study	RC students	n = 89	Pre-workshop, pre-course, post-course and 3-month follow-up

RC, Recovery College; NHS, National Health Service; SWEMWBS, Short Warwick-Edinburgh Mental Wellbeing Scale; SIS, Social Inclusion Scale. Measurement time points are presented as reported in each study.

The included articles were published between 2015 and 2026 and reported research from the United Kingdom (n = 11), Canada (n = 6), Australia (n = 2), Switzerland (n = 1), Ireland (n = 1), and Slovakia (n = 1). Study populations included RC students, adults transitioning from homelessness, NHS staff, and RC stakeholders (14–16). Designs included pre–post and longitudinal evaluations, mixed-methods studies, service evaluations, and cost-benefit analyses (14, 17–20).

Sample sizes and the availability of outcome data varied substantially. Cartier et al. (21) included eight evaluation participants, seven of whom completed questionnaires, whereas Bourne et al. (22) included 463 RC students. Measurement schedules included cross-sectional assessments, immediate post-workshop assessments, and follow-up assessments over subsequent months. Service-use analyses also examined periods extending to 18 months before or after RC registration (14, 16, 21, 22). Further study characteristics are presented in **Table 1**.

### 3.2 Overview of extracted outcome measures

Outcome measures and indicators used in quantitative RC evaluations, including the quantitative components of mixed-methods studies, were extracted. A total of 39 outcome measure and indicator entries were identified, including objective indicators such as health service utilization data derived from clinical or administrative records. These measures assessed personal recovery, well-being, empowerment, self-efficacy, symptoms, stigma, quality of life (QOL), social participation, learning outcomes, and satisfaction, as well as service utilization, economic outcomes, recovery-oriented environments, and recovery-supporting care.

The most frequently used scale was the Warwick-Edinburgh Mental Wellbeing Scale (WEMWBS), including its short version (SWEMWBS), which was used in 11 of the 22 articles (50.0%). The Questionnaire about the Process of Recovery (QPR), the Generalized Anxiety Disorder 7-item scale (GAD-7), and the Opening Minds Stigma Scale for Health Care Providers (OMS-HC/OMS-HCP) were each used in four articles (18.2%). The Empowerment Scale, reported as the Consumer Constructed Scale to Measure Empowerment (CCSME) or the Rogers Making Decisions Empowerment Scale, was used in four articles (18.2%), whereas health service utilization data derived from clinical or administrative records were used in four articles (18.2%) (**Table 2**).

**Table 2:** Overview of extracted outcome measures.

Outcome measure/scale name	Abbreviation	Measured construct	Type	Studies (n)	Reference (author, year, number)
Warwick-Edinburgh Mental Wellbeing Scale (including short form)	WEMWBS/SWEMWBS	Mental well-being	Standardized	11	Allard 2024 (17); Arbour 2023 (29); Briand 2024 (9); Cartier 2026 (21); Ebrahim 2018 (19); Kay 2019 (31); Rapisarda 2022 (26); Rapisarda 2025 (27); Stevens 2018 (23); Wilson 2019 (24); Yoeli 2022 (32)
Questionnaire about the Process of Recovery	QPR	Personal recovery	Standardized	4	Allard 2024 (17); Ambord 2025 (28); Arbour 2023 (29); Durbin 2021 (14)
Health service utilization data from clinical or administrative records	—	Health service utilization	Objective	4	Allard 2024 (17); Bourne 2018 (22); Cronin 2023 (20); Kay 2019 (31)
Generalized Anxiety Disorder 7-item scale	GAD-7	Anxiety symptoms	Standardized	4	Briand 2023 (25); Briand 2024 (9); Rapisarda 2022 (26); Rapisarda 2025 (27)
Opening Minds Stigma Scale for Health Care Providers	OMS-HC/OMS-HCP	Stigma	Standardized	4	Briand 2023 (25); Briand 2024 (9); Rapisarda 2022 (26); Rapisarda 2025 (27)
Empowerment Scale (Consumer Constructed Scale to Measure Empowerment/Rogers Making Decisions Empowerment Scale)	CCSME	Empowerment	Standardized	4	Briand 2024 (9); Rapisarda 2022 (26); Rapisarda 2025 (27); Durbin 2021 (14)
Recovery Assessment Scale	RAS	Personal recovery	Standardized	2	O’Brien 2024 (30); Řezníková 2025 (34)
Connor-Davidson Resilience Scale	CD-RISC-10	Resilience	Standardized	2	Briand 2024 (9); Rapisarda 2025 (27)
Cost-effectiveness/cost-benefit indicators	—	Economic impact	Objective	2	Cronin 2023 (20); Kay 2019 (31)
Mental Health Self-Management Questionnaire	MHSQ	Self-management	Standardized	2	Briand 2023 (25); Briand 2024 (9)
Self-reported health service utilization indicators	—	Reported service contact/perceived reduction in service need	Self-reported indicator	2	Briand 2023 (25); Ebrahim 2018 (19)
Recovery Knowledge Inventory	RKI	Knowledge of recovery	Standardized (wording adapted)	1	O’Brien 2024 (30)
INSPIRE	INSPIRE	Recovery-supporting care/staff support for personal recovery	Standardized	1	Durbin 2021 (14)
CHoice of Outcome in Cbt for Psychoses	CHOICE	Psychological recovery and mental health	Standardized (short form; goal item omitted)	1	Stevens 2018 (23)
WHO-Five Well-Being Index	WHO-5	Psychological well-being	Standardized	1	Ambord 2025 (28)
Quality of Life Inventory-20	QoLi-20	Health-related quality of life	Standardized	1	Durbin 2021 (14)
12-Item Short Form Survey	SF-12	Health-related quality of life	Standardized	1	Durbin 2021 (14)
EuroQol 5-Dimension 5-Level	EQ-5D-5L	Health-related quality of life	Standardized	1	Durbin 2021 (14)
Patient Activation Measure	PAM	Self-management/self-efficacy	Standardized	1	Kay 2019 (31)
Pearlin Mastery Scale	—	Mastery/perceived control	Standardized	1	Durbin 2021 (14)
Self-Stigma of Mental Illness Scale - Short Form	SSMIS-SF	Self-stigma (five-item application subscale only)	Standardized	1	Ambord 2025 (28)
Health of the Nation Outcome Scales	HoNOS	Clinical functioning	Standardized	1	Bourne 2018 (22)
Campaign to End Loneliness Scale	—	Loneliness/connectedness	Standardized	1	Arbour 2023 (29)
Single-item self-esteem scale	—	Self-esteem	Standardized	1	Arbour 2023 (29)
Developing Recovery Enhancing Environments Measure	DREEM	Recovery-oriented environment	Standardized	1	Doroud 2024 (16)
Mind Australia satisfaction survey (modified version)	MASS	Program satisfaction	Service-specific/adapted	1	Doroud 2024 (16)
Client Socio-Demographic and Service Receipt Inventory	CSSRI-EU	Employment, income and health service utilization	Standardized	1	Sutton 2019 (33)
Personal Goal Questionnaire	—	Individual goal attainment	Study-specific	1	Yoeli 2022 (32)
Arts Participation Measure	—	Engagement in arts	Study-specific	1	Stevens 2018 (23)
Study-specific survey of staff experiences and impacts	—	Recovery attitudes, clinical practice, peer interactions and personal well-being	Study-specific	1	Perkins 2017 (15)
Social Inclusion Scale (12-item shortened version)	SIS	Social inclusion: isolation, relations and acceptance	Standardized	1	Wilson 2019 (24)
Empower Flower	—	Personal power and resources across eight domains	Study-specific	1	Ebrahim 2018 (19)
Follow-up questionnaire on RC experiences and future plans (Wilson)	—	Enjoyment, perceived benefits, confidence, motivation and participation intentions	Study-specific	1	Wilson 2019 (24)
Learning-objective achievement questionnaire (Briand)	—	Mental health knowledge, use of tools and perceived learning benefits	Study-specific	1	Briand 2023 (25)
Course-feedback questionnaire (Ebrahim)	—	Course experience, confidence, motivation, recommendation and perceived service-use change	Study-specific	1	Ebrahim 2018 (19)
Feelings chart (Cartier)	—	Current emotions before and after the workshop	Study-specific	1	Cartier 2026 (21)
Post-workshop appraisal and goal-achievement questionnaire (Cartier)	—	Helpfulness, enjoyment, perceived change and attainment of self-identified goals	Study-specific	1	Cartier 2026 (21)
Pre/post self-rating questionnaire (Burhouse)	—	Hope, recovery, knowledge/self-awareness and self-management skills	Study-specific	1	Burhouse 2015 (18)
Manhattan Recovery Measure	—	Personal recovery and progress toward individualized recovery goals	Previously developed; common and individualized items; validation status not reported	1	Burhouse 2015 (18)

Counts represent the number of included articles using each measure or indicator (22 articles overall); each article is counted once per row, irrespective of repeated assessments or subscales. Full and short versions are combined. Study-specific questionnaires are retained separately unless they are the same instrument. This table contains 39 inventory entries, not 39 independent standardized scales. Some indicator rows overlap with questionnaire rows: the self-reported service-use item in Ebrahim (2018) (19) is also part of its course-feedback questionnaire. Therefore, counts across rows should not be summed. “Standardized” identifies previously developed instruments and does not establish their measurement quality in RC settings. “Objective” identifies clinical/administrative or economic data; self-reported service use is shown separately. Demographics, contextual COVID-19 stress and qualitative data-collection methods are not counted as outcome instruments.

The contexts and measurement schedules in which these instruments were used varied. In UK studies, WEMWBS/SWEMWBS was used in evaluations of general RC provision, arts courses, and a Feldenkrais and vocal improvisation workshop (17, 21, 23). Wilson et al. (24) used SWEMWBS alongside the Social Inclusion Scale at baseline and at three- and six-month follow-up, whereas Cartier et al. (21) administered SWEMWBS before the workshop and at two-week follow-up. In Canadian online RC evaluations, GAD-7 and OMS-HC/OMS-HCP were used within repeated-measures designs incorporating baseline, post-intervention, and subsequent follow-up assessments (9, 25–27). QPR was used to assess personal recovery in populations including general RC students and adults transitioning from homelessness (14, 17, 28, 29). Cross-sectional evaluations also used measures for different purposes: Doroud et al. (16) used the Developing Recovery Enhancing Environments Measure (DREEM) and a modified Mind Australia satisfaction survey (MASS) to assess the recovery-supporting environment and program satisfaction in an Australian RC, while O’Brien et al. (30) used the Recovery Assessment Scale (RAS) and Recovery Knowledge Inventory (RKI) to assess personal recovery and recovery-related knowledge in an Irish recovery education evaluation. Service-use and economic evaluations incorporated clinical or administrative records and cost-related indicators (20, 22, 31).

Of the 39 inventory entries, 28 were used in only one article. Alongside previously developed instruments, studies used study-specific questionnaires and indicators assessing personal goal attainment, learning-objective achievement, personal resources, current emotions, and course experiences (19, 21, 25, 32). Burhouse et al. (18) used a pre/post self-rating questionnaire covering hope, recovery, knowledge/self-awareness, and self-management, together with the Manhattan Recovery Measure, which combined common questions with individualized questions.

Self-reported service-use indicators were distinguished from utilization data obtained from clinical or administrative records (19, 25). The inventory entries were not necessarily independent instruments: for example, the service-use item in Ebrahim et al. (19) also formed part of the course-feedback questionnaire. Full and short versions of the same instrument were counted together, whereas study-specific questionnaires were retained separately unless they were identified as the same instrument (**Table 2**).

### 3.3 Classification of measured constructs

The measured constructs were grouped into three major categories: (1) intra-personal and psychological changes, (2) behavioral and social changes, and (3) system and environment (**Table 3**). These categories contained 26, seven, and six inventory entries, respectively. The categories were defined according to the principal object of measurement, rather than the identity of the respondent or the data-collection method. Each inventory entry was assigned to one category according to its principal measured content. The term “changes” in the category labels denotes the outcome domain and does not imply that every included study assessed change over time.

**Table 3:** Classification of outcome measures by measured constructs.

Major category	Subcategory	Specific construct	Outcome measure/scale name (abbreviation)	Brief description of measured content
(1) Intra-personal and psychological changes	① Recovery and its process	Recovery process	Recovery Assessment Scale (RAS) (30, 34)	Assesses personal recovery.
(1) Intra-personal and psychological changes	① Recovery and its process	Recovery process	Questionnaire about the Process of Recovery (QPR) (14, 17, 28, 29)	Assesses personal recovery.
(1) Intra-personal and psychological changes	① Recovery and its process	Recovery process	Pre/post self-rating questionnaire (Burhouse) (18)	Assesses hope and recovery, knowledge/self-awareness and self-management before and after RC attendance. Includes confidence, self-control, routines and understanding of others.
(1) Intra-personal and psychological changes	① Recovery and its process	Recovery process	Manhattan Recovery Measure (18)	Combines common questions with four individually designed questions to track recovery across the course. Personal goal progress is reported; detailed scoring and attribution of individual results are not fully specified.
(1) Intra-personal and psychological changes	① Recovery and its process	Knowledge and understanding of recovery	Recovery Knowledge Inventory (RKI) (30)	Assesses knowledge of recovery.
(1) Intra-personal and psychological changes	① Recovery and its process	Psychological recovery	CHoice of Outcome in Cbt for Psychoses (CHOICE) (23)	Assesses psychological recovery and mental health using the short form, with the personal-goal item omitted.
(1) Intra-personal and psychological changes	① Recovery and its process	Goal attainment	Personal Goal Questionnaire (32)	Assesses individual goal attainment.
(1) Intra-personal and psychological changes	② Empowerment and subjective control	Self-management	Mental Health Self-Management Questionnaire (MHSQ) (9, 25)	Assesses self-management.
(1) Intra-personal and psychological changes	② Empowerment and subjective control	Self-management	Patient Activation Measure (PAM) (31)	Assesses self-management/self-efficacy.
(1) Intra-personal and psychological changes	② Empowerment and subjective control	Empowerment	Empowerment Scale (CCSME/Rogers Making Decisions Empowerment Scale) (9, 14, 26, 27)	Assesses empowerment; instrument versions and names are consolidated, while study-specific reporting is retained in **Table 4**.
(1) Intra-personal and psychological changes	② Empowerment and subjective control	Personal power and resources	Empower Flower (19)	Assesses perceived personal power and resources across eight domains using the study-specific Empower Flower.
(1) Intra-personal and psychological changes	② Empowerment and subjective control	Self-esteem	Single-item self-esteem scale (29)	Assesses self-esteem.
(1) Intra-personal and psychological changes	② Empowerment and subjective control	Resilience	Connor-Davidson Resilience Scale (CD-RISC-10) (9, 27)	Assesses resilience.
(1) Intra-personal and psychological changes	② Empowerment and subjective control	Mastery	Pearlin Mastery Scale (14)	Assesses perceived mastery and control over life.
(1) Intra-personal and psychological changes	③ Well-being and QOL	Well-being	Warwick-Edinburgh Mental Wellbeing Scale (including short form) (WEMWBS/SWEMWBS) (9, 17, 19, 21, 23, 24, 26, 27, 29, 31, 32)	Assesses mental well-being.
(1) Intra-personal and psychological changes	③ Well-being and QOL	Well-being	WHO-Five Well-Being Index (WHO-5) (28)	Assesses psychological well-being.
(1) Intra-personal and psychological changes	③ Well-being and QOL	Well-being	Study-specific survey of staff experiences and impacts (15)	Assesses perceived changes in recovery attitudes, clinical practice, peer interactions and personal well-being among staff.
(1) Intra-personal and psychological changes	③ Well-being and QOL	Health-related QOL	Quality of Life Inventory-20 (QoLi-20) (14)	Assesses health-related quality of life.
(1) Intra-personal and psychological changes	③ Well-being and QOL	Health-related QOL	12-Item Short Form Survey (SF-12) (14)	Assesses health-related quality of life.
(1) Intra-personal and psychological changes	③ Well-being and QOL	Health-related QOL	EuroQol 5-Dimension 5-Level (EQ-5D-5L) (14)	Assesses health-related quality of life.
(1) Intra-personal and psychological changes	④ Symptoms and emotional states	Symptoms and clinical functioning	Generalized Anxiety Disorder 7-item scale (GAD-7) (9, 25–27)	Assesses anxiety symptoms.
(1) Intra-personal and psychological changes	④ Symptoms and emotional states	Symptoms and clinical functioning	Health of the Nation Outcome Scales (HoNOS) (22)	Assesses clinical functioning.
(1) Intra-personal and psychological changes	④ Symptoms and emotional states	Current emotional state	Feelings chart (Cartier) (21)	Records current positive and negative emotions before and after a workshop using a study-specific feelings chart; does not assess symptom severity.
(1) Intra-personal and psychological changes	⑤ Stigma-related attitudes and self-stigma	Stigma	Opening Minds Stigma Scale for Health Care Providers (OMS-HC/OMS-HCP) (9, 25–27)	Assesses stigma.
(1) Intra-personal and psychological changes	⑤ Stigma-related attitudes and self-stigma	Stigma	Self-Stigma of Mental Illness Scale - Short Form (SSMIS-SF) (28)	Assesses application of self-stigma using five items from the SSMIS-SF.
(1) Intra-personal and psychological changes	⑥ Learning outcomes	Mental health knowledge and skills	Learning-objective achievement questionnaire (Briand) (25)	Assesses achievement of learning objectives, including mental health knowledge, use of intervention tools and perceived learning benefits.
(2) Behavioral and social changes	① Health service utilization and health economics	Health service utilization	Health service utilization data from clinical or administrative records (17, 20, 22, 31)	Assesses health service utilization.
(2) Behavioral and social changes	① Health service utilization and health economics	Health service utilization	Self-reported health service utilization indicators (19, 25)	Captures self-reported service contacts or perceived reductions in service need; distinct from utilization obtained from clinical or administrative records.
(2) Behavioral and social changes	① Health service utilization and health economics	Employment, income and service receipt	Client Socio-Demographic and Service Receipt Inventory (CSSRI-EU) (33)	Assesses employment, income and health service utilization.
(2) Behavioral and social changes	① Health service utilization and health economics	Economic impact	Cost-effectiveness/cost-benefit indicators (20, 31)	Assesses economic impact.
(2) Behavioral and social changes	② Social connectedness	Arts participation and connectedness	Arts Participation Measure (23)	Assesses engagement in arts.
(2) Behavioral and social changes	② Social connectedness	Loneliness	Campaign to End Loneliness Scale (29)	Assesses loneliness/connectedness.
(2) Behavioral and social changes	② Social connectedness	Social inclusion	Social Inclusion Scale (12-item shortened version) (SIS) (24)	Assesses social inclusion through social isolation, social relations and social acceptance using a shortened 12-item scale.
(3) System and environment	① Program appraisal and experience	Perceived program benefits and acceptability	Follow-up questionnaire on RC experiences and future plans (Wilson) (24)	Assesses enjoyment and perceived benefits of RC attendance, confidence, motivation and intentions for future participation.
(3) System and environment	① Program appraisal and experience	Perceived program benefits and acceptability	Course-feedback questionnaire (Ebrahim) (19)	Assesses course experience, confidence, motivation, willingness to recommend the course and perceived change in service use.
(3) System and environment	① Program appraisal and experience	Perceived program benefits and acceptability	Post-workshop appraisal and goal-achievement questionnaire (Cartier) (21)	Assesses workshop helpfulness and enjoyment, perceived change and retrospective attainment of self-identified goals.
(3) System and environment	① Program appraisal and experience	RC satisfaction	Mind Australia satisfaction survey (modified version) (MASS) (16)	Assesses program satisfaction.
(3) System and environment	② Recovery-oriented environment	Environmental evaluation	Developing Recovery Enhancing Environments Measure (DREEM) (16)	Assesses the recovery-enhancing environment using the Developing Recovery Enhancing Environments Measure.
(3) System and environment	③ Recovery-supporting care	Recovery-supporting care	INSPIRE (14)	Assesses recovery-supporting care/staff support for personal recovery.

Each of the 39 inventory entries in **Table 2** is assigned to one domain according to its principal measured content. Full and short forms are combined. For questionnaires covering several concepts, the assigned domain does not encompass every item. Program-appraisal questionnaires include individual-level benefits and goal attainment alongside course experience. Domain totals therefore describe inventory entries, not independent scales or numbers of studies. Classification does not indicate measurement quality. QOL, quality of life; RC, Recovery College. Within each major category, subcategories are ordered by the number of inventory entries, and specific constructs are grouped and ordered similarly within each subcategory. Ties retain their previous relative order.

**Table 3** continued — Definitions of categories and subcategories

Categories are defined by the principal object of measurement, rather than respondent identity or data-collection method. Each inventory entry is assigned to one category. “Changes” denotes an outcome domain and does not imply that every study assessed change over time.

(1) Intra-personal and psychological changes. Individuals’ internal experiences, knowledge, attitudes, perceived capacities, and mental health status, including personal recovery and psychological functioning.

① Recovery and its process: Personal recovery, understanding of recovery, and progress toward self-defined recovery goals.

② Empowerment and subjective control: Perceived agency, personal resources, self-worth, resilience, and capacity to manage one’s health and life.

③ Well-being and QOL: Mental well-being and perceived quality of life, including health-related quality of life.

④ Symptoms and emotional states: Mental health symptoms, associated clinical or functional difficulties, and current positive and negative emotional states. Measures of current emotions do not necessarily assess symptom severity.

⑤ Stigma-related attitudes and self-stigma: Stigmatizing beliefs and attitudes concerning mental illness, including internalized stigma and attitudes toward social distance, disclosure, or help-seeking.

⑥ Learning outcomes: Knowledge and skills addressed through RC education, including perceived achievement of learning objectives and reported use of tools introduced through learning.

(2) Behavioral and social changes. Participation in activities and social life, relationships and social circumstances, and patterns of health service use and their economic consequences. This includes reported experiences of social connection as well as participation and service-use indicators.

① Health service utilization and health economics: Contacts with health services, reported need for services, employment and income-related circumstances, and costs or economic consequences associated with service use and RC participation.

② Social connectedness: Participation in shared activities, relationships with others, social inclusion and acceptance, and experiences of loneliness or isolation.

(3) System and environment. Characteristics of RC provision and the surrounding recovery-supporting context, together with participants’ appraisals of the program and support received. The object of evaluation is the environment, provision, or support, even when individuals report the information.

① Program appraisal and experience: Participants’ evaluations of RC provision, including satisfaction, acceptability, helpfulness, enjoyment, and perceived program benefits.

② Recovery-oriented environment: The extent to which the learning or service environment is experienced as supporting recovery.

③ Recovery-supporting care: The extent to which staff support individuals’ personal recovery and recovery goals.

The categories are conceptually related rather than mutually exclusive at the item level. Stigma measures include interpersonal attitudes, clinical functioning measures may include social functioning, and program-appraisal questionnaires may include confidence, motivation, or goal attainment. Assignment follows the principal measured content; additional content is retained in the measure descriptions. Placement in the system and environment category does not imply direct measurement of organizational change, and individual-level placement does not exclude social influences.

#### 3.3.1 Intra-personal and psychological changes

This category encompasses individuals’ internal experiences, knowledge, attitudes, perceived capacities, and mental health status, including personal recovery and psychological functioning. It includes six subcategories:

##### 3.3.1.1 Recovery and its process

This subcategory encompasses personal recovery, understanding of recovery, and progress toward self-defined recovery goals. Measures included the Recovery Assessment Scale (RAS) and QPR for personal recovery, the Recovery Knowledge Inventory (RKI) for recovery-related knowledge, CHOICE for psychological recovery and mental health, and the Personal Goal Questionnaire for individual goal attainment (14, 23, 30, 32). Burhouse et al. (18) used a pre/post self-rating questionnaire covering hope, recovery, knowledge/self-awareness, and self-management, together with the Manhattan Recovery Measure to track personal recovery. The latter included common questions and four individually designed questions; detailed scoring and the attribution of individual outcome results to the measures were not fully specified.

##### 3.3.1.2 Empowerment and subjective control

This subcategory encompasses perceived agency, personal resources, self-worth, resilience, and capacity to manage one’s health and life. Measures included the Empowerment Scale (CCSME/Rogers Making Decisions Empowerment Scale), Empower Flower, the single-item self-esteem scale, CD-RISC-10, MHSQ, PAM, and the Pearlin Mastery Scale (9, 14, 19, 29, 31).

##### 3.3.1.3 Well-being and QOL

This subcategory encompasses mental well-being and perceived quality of life, including health-related quality of life. Measures included WEMWBS/SWEMWBS and WHO-5 for well-being, and QoLi-20, SF-12, and EQ-5D-5L for quality of life. Study-specific assessments also addressed staff experiences and perceived impacts (14, 15, 17, 28).

##### 3.3.1.4 Symptoms and emotional states

This subcategory encompasses mental health symptoms, associated clinical or functional difficulties, and current positive and negative emotional states. GAD-7 assessed anxiety symptoms, HoNOS assessed clinical and social functioning, and the Feelings Chart assessed current emotional states (21, 22, 26). Measures of current emotions do not necessarily assess symptom severity.

##### 3.3.1.5 Stigma-related attitudes and self-stigma

This subcategory encompasses stigmatizing beliefs and attitudes concerning mental illness, including internalized stigma and attitudes toward social distance, disclosure, or help-seeking. OMS-HC/OMS-HCP assessed stigma-related attitudes, while the five-item application subscale of the Self-Stigma of Mental Illness Scale–Short Form (SSMIS-SF) assessed the application of self-stigmatizing beliefs (25, 26, 28).

##### 3.3.1.6 Learning outcomes

This subcategory encompasses knowledge and skills addressed through RC education, including perceived achievement of learning objectives and reported use of tools introduced through learning. A study-specific questionnaire assessed mental health knowledge, use of intervention tools, and perceived learning benefits (25).

#### 3.3.2 Behavioral and social changes

This category encompasses participation in activities and social life, relationships and social circumstances, and patterns of health service use and their economic consequences. It includes reported experiences of social connection as well as participation and service-use indicators. Two subcategories were identified:

##### 3.3.2.1 Health service utilization and health economics

This subcategory encompasses contacts with health services, reported need for services, employment and income-related circumstances, and costs or economic consequences associated with service use and RC participation. Indicators included service-use data obtained from clinical or administrative records, self-reported service-use indicators, the Client Socio-Demographic and Service Receipt Inventory (CSSRI-EU), and cost-effectiveness or cost-benefit indicators. CSSRI-EU also captured employment and income-related information (19, 20, 22, 25, 31, 33).

##### 3.3.2.2 Social connectedness

This subcategory encompasses participation in shared activities, relationships with others, social inclusion and acceptance, and experiences of loneliness or isolation. Measures included the Arts Participation Measure, the Campaign to End Loneliness Scale, and the Social Inclusion Scale (23, 24, 29).

#### 3.3.3 System and environment

This category encompasses the characteristics of RC provision and the surrounding recovery-supporting context, together with participants’ appraisals of the program and support received. The object of evaluation is the environment, provision, or support, even when individuals report the information. Three subcategories were identified:

##### 3.3.3.1 Program appraisal and experience

This subcategory encompasses participants’ evaluations of RC provision, including satisfaction, acceptability, helpfulness, enjoyment, and perceived program benefits. Measures included the modified Mind Australia satisfaction survey (MASS) and study-specific questionnaires addressing course experiences, perceived benefits, and acceptability (16, 19, 21, 24).

##### 3.3.3.2 Recovery-oriented environment

This subcategory concerns the extent to which the learning or service environment is experienced as supporting recovery. The Developing Recovery Enhancing Environments Measure (DREEM) assessed the recovery-enhancing environment (16).

##### 3.3.3.3 Recovery-supporting care

This subcategory concerns the extent to which staff support individuals’ personal recovery and recovery goals. INSPIRE assessed perceived staff support for personal recovery (14).

The categories are conceptually related rather than mutually exclusive at the item level. For example, stigma measures include interpersonal attitudes, clinical functioning measures may include social functioning, and program-appraisal questionnaires may include confidence, motivation, or personal goal attainment. Assignment followed the principal measured content, while additional content was retained in the descriptions in **Table 3**. Placement in the system and environment category does not imply direct measurement of organizational change, and placement in an individual-level category does not exclude social influences on the measured construct.

### 3.4 Reporting status of reliability and validity

An examination of reliability and validity reporting distinguished assessments conducted within the included studies from references to previously published psychometric information (**Table 4**). **Table 4** covers 37 questionnaire and indicator entries; the two aggregate entries for record-derived service use and cost indicators were excluded from scale psychometrics. Of the 22 included articles, 15 (68.2%) cited previous psychometric information or identifiable validation literature for at least one extracted measure. Two articles (9.1%) explicitly reported reliability estimates calculated from their own study data (26, 30).

**Table 4:** Reporting status of reliability and validity.

Outcome measure/indicator	Within-study reliability	Previously published psychometric information	Within-study validity information	Version and reporting notes
Warwick-Edinburgh Mental Wellbeing Scale (including short form) (WEMWBS/SWEMWBS) Allard 2024 (17); Arbour 2023 (29); Briand 2024 (9); Kay 2019 (31); Rapisarda 2022 (26); Rapisarda 2025 (27); Stevens 2018 (23); Yoeli 2022 (32); Wilson 2019 (24); Ebrahim 2018 (19); Cartier 2026 (21)	Rapisarda 2022 (26): α = 0.80.	Validation/development or psychometric sources cited: Allard 2024 (17); Briand 2024 (9); Rapisarda 2022 (26); Rapisarda 2025 (27); Stevens 2018 (23); Yoeli 2022 (32); Wilson 2019 (24); Ebrahim 2018 (19); Cartier 2026 (21). Briand 2024 (9) describes α = 0.89–0.91, test–retest = 0.83 and a single-factor structure. Allard 2024 (17) cites prior responsiveness evidence for SWEMWBS.	Not reported.	Arbour 2023 (29) cites prior use and score interpretation; Kay 2019 (31) reports no reliability/validity evidence. Allard 2024 (17) discusses 1–3-point SWEMWBS changes using detectability and clinical-significance terminology; Yoeli 2022 (32) cites a 3-point MCID for 14-item WEMWBS. These thresholds were not newly estimated in the RC samples and are not interchangeable across versions. Full-form evidence does not automatically apply to a short form.
Questionnaire about the Process of Recovery (QPR) Allard 2024 (17); Ambord 2025 (28); Arbour 2023 (29); Durbin 2021 (14)	Not reported.	Allard 2024 (17) and Durbin 2021 (14) cite psychometric evaluations. Ambord 2025 (28) describes German 15-item QPR α = 0.91, corrected item correlations 0.49–0.73 and model-fit evidence in the context of prior validation. Arbour 2023 (29) cites development research and a review mapping QPR items to CHIME.	Not reported.	No clearly identified new reliability/validity analysis. Ambord (28) names RMSEA, CFI and TLI without numerical indices. Cited change thresholds include 5 points within persons and 4 points between groups for a specific clinical population (Allard 2024) (17), and 5 points within persons (Ambord 2025) (28). These were not newly established in the RC studies; CHIME mapping describes content coverage.
Generalized Anxiety Disorder 7-item scale (GAD-7) Briand 2024 (9); Rapisarda 2022 (26); Rapisarda 2025 (27); Briand 2023 (25)	Rapisarda 2022 (26): α = 0.87.	Briand 2023 (25): α = 0.92, cited. Briand 2024 (9): α = 0.92, test–retest = 0.83, single-factor structure, sensitivity 89% and specificity 82% at cut-off 10 described with source citation. Rapisarda 2022 (26); Rapisarda 2025 (27) cite validation sources.	Not reported.	Coefficients in Briand 2023 (25) and Briand 2024 (9) describe prior instrument properties; not identified as newly estimated.
Opening Minds Stigma Scale for Health Care Providers (OMS-HC/OMS-HCP) Briand 2024 (9); Rapisarda 2022 (26); Rapisarda 2025 (27); Briand 2023 (25)	Rapisarda 2022 (26): α = 0.81.	Briand 2023 (25): subscale α = 0.68, 0.67, 0.68, cited. Briand 2024 (9): overall α = 0.79 and subscale α = 0.68, 0.67, 0.68; three-factor structure described. Rapisarda 2022 (26); Rapisarda 2025 (27) cite validation sources.	Not reported.	Subscale labels differ across articles.
Empowerment Scale (CCSME/Rogers Making Decisions Empowerment Scale) Briand 2024 (9); Rapisarda 2022 (26); Rapisarda 2025 (27); Durbin 2021 (14)	Rapisarda 2022 (26): α = 0.77.	Briand 2024 (9): subscale α = 0.82, 0.59, 0.59, 0.45, 0.64; five-factor structure described. Rapisarda 2022 (26) and Rapisarda 2025 (27) cite prior instrument literature. Durbin 2021 (14) reports prior α = 0.86 and factorial validity.	Not reported.	Rapisarda 2025 (27) reports internal consistency = 0.84 without clearly identifying the data source; provenance unclear, not counted as confirmed within-study reliability. Durbin (14) cites Rogers et al. (1997) for the measure, as does Briand 2024 (9). The adjacent citation in Durbin (14) for psychometric information points to a quality-of-life paper; citation alignment remains unclear. Reported values are retained without attributing them to the current sample.
Recovery Assessment Scale (RAS) O’Brien 2024 (30); Řezníková 2025 (34)	O’Brien 2024 (30): Cronbach’s α = 0.93. Řezníková 2025 (34): not reported.	O’Brien 2024 (30) describes the RAS as well validated and cites factor-structure research and a review of its reliability and validity. Řezníková 2025 (34) describes five dimensions and cites a review of recovery measures, without specific psychometric results.	O’Brien 2024 (30) states that convergent and divergent validity tests were conducted. Communalities > 0.5 and KMO = 0.8 are reported in the paragraph addressing RAS and RKI. Řezníková 2025 (34): not reported.	The specific validity-testing procedures and the allocation of the communalities and KMO statistic to RAS versus RKI are not clearly reported.
Connor-Davidson Resilience Scale (CD-RISC-10) Briand 2024 (9); Rapisarda 2025 (27)	Unclear for Rapisarda 2025 (27); see notes.	Briand 2024 (9): α = 0.85, test–retest = 0.81 and single-factor structure described. Rapisarda 2025 (27) cites the original instrument literature.	Not reported.	Rapisarda 2025 (27) reports α = 0.85 without clearly identifying whether it was estimated in the current sample. Provenance unclear.
Mental Health Self-Management Questionnaire (MHSQ) Briand 2024 (9); Briand 2023 (25)	Not reported.	Briand 2023 (25) cites subscale internal-consistency coefficients of 0.69, 0.81 and 0.75. Briand 2024 (9) describes Clinical α = 0.69, Empowerment α = 0.81 and Vitality α = 0.75, with test–retest coefficients of 0.78, 0.76 and 0.85, respectively, and a three-factor structure.	Not reported.	Prior instrument information; no explicit current-sample estimation. Briand 2024 (9) labels the test–retest coefficients with α; the coefficient type is not clarified. Subscale terminology differs between Briand 2023 (25) and Briand 2024 (9).
Self-reported health service utilization indicators Briand 2023 (25); Ebrahim 2018 (19)	Not reported.	Not reported.	Not reported.	No reliability/validity assessment reported.
Recovery Knowledge Inventory (RKI) O’Brien 2024 (30)	O’Brien 2024 (30): α = 0.75.	O’Brien 2024 (30) describes the RKI as validated and cites its source.	O’Brien 2024 (30) states that convergent and divergent validity tests were conducted in a paragraph addressing RAS and RKI; communalities > 0.5 and KMO = 0.8 are reported without an explicit scale-specific allocation.	Wording was adapted to reflect contemporary recovery language. Specific validity-testing procedures and scale-specific statistics are not clearly reported. The shared RAS/RKI paragraph is counted once at article level.
INSPIRE Durbin 2021 (14)	Not reported.	Durbin 2021 (14) cites the development and evaluation of INSPIRE.	Not reported.	No coefficient or new analysis reported for INSPIRE in the included study.
CHoice of Outcome in Cbt for Psychoses (CHOICE) Stevens 2018 (23)	Not reported.	Stevens 2018 (23) states that the original CHOICE had good psychometric properties and cites its source.	Not reported.	The short form was used with personal-goal item 12 omitted. The article explicitly states that no psychometric information was available for the short form.
WHO-Five Well-Being Index (WHO-5) Ambord 2025 (28)	Not reported.	Ambord 2025 (28) describes prior validation of the German translation (Brähler et al., 2007), including α = 0.92, and cites a suggested 10% mean-score difference for clinical relevance (Topp et al., 2015).	No clearly identified current-sample validity analysis. The measure description states that model fit was good on RMSEA, CFI and TLI.	Numerical model-fit indices and the data source for the model-fit statement are not specified. The cited α and change-interpretation threshold should not be treated as newly estimated in this RC sample.
Quality of Life Inventory-20 (QoLi-20) Durbin 2021 (14)	Not reported.	Durbin 2021 (14) cites a graded-response modelling study of the quality-of-life instrument.	Not reported.	No reliability coefficient or new psychometric analysis reported; evidence for the exact 20-item form is not established merely by the citation.
12-Item Short Form Survey (SF-12) Durbin 2021 (14)	Not reported.	Durbin 2021 (14) cites the SF-12 construction and preliminary reliability/validity study.	Not reported.	Citation present; no within-study reliability/validity analysis reported.
EuroQol 5-Dimension 5-Level (EQ-5D-5L) Durbin 2021 (14)	Not reported.	Not reported.	Not reported.	Durbin 2021 (14) cites the instrument website; no specific reliability/validity evidence reported.
Patient Activation Measure (PAM) Kay 2019 (31)	Not reported.	Not reported.	Not reported.	Kay 2019 (31) reports PAM outcomes without reliability/validity information.
Pearlin Mastery Scale Durbin 2021 (14)	Not reported.	Not reported.	Not reported.	Durbin 2021 (14) cites the original coping source but reports no specific reliability/validity information for mastery.
Self-Stigma of Mental Illness Scale - Short Form (SSMIS-SF) Ambord 2025 (28)	Not reported.	Ambord 2025 (28): previous subsample α values of 0.74, 0.69 and 0.22 for the application-of-self-stigma subscale.	Not reported.	Only the five-item application subscale was used. The article states that the German SSMIS-SF had not yet been validated.
Health of the Nation Outcome Scales (HoNOS) Bourne 2018 (22)	Not reported.	Not reported.	Not reported.	Bourne 2018 (22) reports HoNOS outcomes and excludes an invalid score; this is data cleaning, not psychometric evaluation.
Campaign to End Loneliness Scale Arbour 2023 (29)	Not reported.	Not reported.	Not reported.	Arbour 2023 (29) describes three items and co-design but reports no reliability/validity evidence.
Single-item self-esteem scale Arbour 2023 (29)	Not reported.	Arbour 2023 (29) cites predictive and convergent validity of the single-item measure relative to the Rosenberg scale.	Not reported.	No within-study reliability/validity analysis reported.
Developing Recovery Enhancing Environments Measure (DREEM) Doroud 2024 (16)	Not reported.	Doroud 2024 (16) describes DREEM as a validated instrument and cites previous sources.	Not reported.	Fourteen closed questions were used; no new psychometric evaluation of this item set is reported. The instrument concerns recovery-enhancing environments.
Mind Australia satisfaction survey (modified version) (MASS) Doroud 2024 (16)	Not reported.	Not reported.	Not reported.	Doroud 2024 (16) uses modified MASS; no reliability/validity evaluation reported for the modified survey.
Client Socio-Demographic and Service Receipt Inventory (CSSRI-EU) Sutton 2019 (33)	Not reported.	Not reported.	Not reported.	Sutton 2019 (33) cites the original CSSRI-EU development paper and describes consultation on wording and burden. No psychometric results for the modified item set are reported; source citation alone is not coded as a validation citation.
Personal Goal Questionnaire Yoeli 2022 (32)	Not reported.	Not reported.	Not reported.	Yoeli 2022 (32) describes co-development and scoring; no reliability/validity assessment reported.
Arts Participation Measure Stevens 2018 (23)	Not reported.	Not reported.	Stevens 2018 (23) states that face and content validity were established; measure designed in consultation with service users.	Non-statistical validity statement; no coefficients or formal testing protocol reported. Not counted as statistical validation within the study sample.
Study-specific survey of staff experiences and impacts Perkins 2017 (15)	Not reported.	Not reported.	Not reported.	Perkins 2017 (15) reports survey development and outcomes but no reliability/validity evidence.
Social Inclusion Scale (12-item shortened version) (SIS) Wilson 2019 (24)	Not reported.	Wilson 2019 (24): original SIS reliability/validity literature cited; internal consistency of the shortened 12-item form cited separately.	Not reported.	No numeric coefficient or new psychometric analysis reported.
Empower Flower Ebrahim 2018 (19)	Not reported.	Not reported.	Ebrahim 2018 (19) describes participant feedback on relevance/ease of use and refers to face validity.	Informal face-validity information only. The article explicitly states that reliability/validity testing had not been undertaken; this is distinct from formal validation.
Follow-up questionnaire on RC experiences and future plans (Wilson) Wilson 2019 (24)	Not reported.	Not reported.	Not reported.	No reliability/validity assessment reported.
Learning-objective achievement questionnaire (Briand) Briand 2023 (25)	Not reported.	Not reported.	Not reported.	No reliability/validity assessment reported.
Course-feedback questionnaire (Ebrahim) Ebrahim 2018 (19)	Not reported.	Not reported.	Not reported.	No reliability/validity assessment reported.
Feelings chart (Cartier) Cartier 2026 (21)	Not reported.	Not reported.	Not reported.	No reliability/validity assessment reported.
Post-workshop appraisal and goal-achievement questionnaire (Cartier) Cartier 2026 (21)	Not reported.	Not reported.	Not reported.	No reliability/validity assessment reported.
Pre/post self-rating questionnaire (Burhouse) Burhouse 2015 (18)	Not reported.	Not reported.	Not reported.	Study-specific self-ratings of hope, recovery, knowledge/self-awareness and self-management. Item wording, response options and scoring are not fully reported.
Manhattan Recovery Measure Burhouse 2015 (18)	Not reported.	Burhouse 2015 (18) cites Davies (2011) as the instrument source; no specific reliability/validity evidence reported.	Not reported.	Common questions plus four individualized questions. The source citation alone does not establish validation. Detailed scoring and outcome-to-instrument attribution are incompletely reported.

The 37 questionnaire/indicator entries follow **Table 2** order; its two aggregate entries for record-derived service use and costs are excluded from scale psychometrics. “Not reported” means no identified reporting, not poor measurement quality. Prior information includes explicit psychometric descriptions and identifiable validation/evaluation citations; cited papers were not independently assessed. A generic instrument-source citation is insufficient. Current-sample reliability requires explicit attribution; unclear provenance is flagged. Non-statistical face/content-validity descriptions are distinguished from statistical testing. Evidence is version-specific and is not automatically transferable. Cited responsiveness and change-interpretation thresholds are contextual information, not new RC-specific validation. Internal consistency may not apply to single items, individualized goals or heterogeneous checklists. Article-level percentages use 22 articles with overlapping categories; Cronin (2023) (20) is not applicable to scale psychometrics. MCID, minimum clinically important difference; KMO, Kaiser–Meyer–Olkin measure of sampling adequacy; RMSEA, root mean square error of approximation; CFI, comparative fit index; TLI, Tucker–Lewis index.

Reference numbers in parentheses correspond to the manuscript reference list.

Rapisarda et al. (26) reported Cronbach’s α values of 0.87 for GAD-7, 0.81 for OMS-HC, 0.80 for SWEMWBS, and 0.77 for CCSME. O’Brien et al. (30) reported α = 0.93 for RAS and α = 0.75 for RKI. One additional article (4.5%) reported reliability coefficients without clearly identifying whether they were estimated in the current sample or obtained from previous research; these were retained as having unclear provenance rather than counted as confirmed within-study estimates (27).

One article (4.5%) reported undertaking statistical assessments of validity within the study (30). The authors stated that convergent and divergent validity testing had been conducted and reported communalities above 0.5 and a Kaiser–Meyer–Olkin value of 0.8, although the allocation of these statistics to individual scales was not fully specified. Two further articles (9.1%) included non-statistical descriptions concerning face or content validity: Stevens et al. (23) stated that face and content validity had been established for the Arts Participation Measure, while Ebrahim et al. (19) described participant feedback supporting the face validity of Empower Flower but explicitly noted that formal reliability and validity testing had not been undertaken.

Six articles (27.3%) provided neither identifiable reliability or validity evidence nor a specific validation citation for the extracted measures (15, 18, 22, 31, 33, 34). Burhouse et al. (18) cited the source of the Manhattan Recovery Measure but reported no specific reliability or validity evidence; this source citation was not classified as a validation citation. One article (4.5%), a record-based service-use and cost-benefit analysis, was classified separately as not applicable to scale psychometrics (20). All percentages use the 22 included articles as the denominator. Reporting categories overlap because an article could report more than one type of information.

### 3.5 Main purposes of using indicators in RC research

Outcome measures were used to evaluate psychological changes, such as personal recovery, well-being, empowerment, and stigma, before and after program participation (9, 17, 26, 28). Some studies incorporated subsequent follow-up assessments to examine outcomes beyond the immediate post-intervention period (14, 24, 25). Measures were also used to examine differences in individual patterns of change across several outcome dimensions (27).

In addition, indicators were used to assess learning-objective achievement, personal goal attainment, and participation in social or creative activities (23–25, 32). Burhouse et al. (18) used self-report measures to evaluate hope, recovery-related knowledge, self-management, and progress toward personal recovery goals within a quality-improvement project. Studies focusing on service use and economic outcomes examined contacts with health services, hospital admissions, employment-related outcomes, and potential cost savings associated with RC participation (20, 22, 31, 33).

Other measures were used to assess recovery-supporting environments, perceived staff support, satisfaction, and participants’ experiences of RC provision (14, 16, 19, 21). O’Brien et al. (30) used cross-sectional quantitative data to evaluate personal recovery and recovery-related knowledge in the context of recovery education. Thus, the purposes of measurement included both the assessment of participant outcomes and the evaluation of RC provision, and were not limited to longitudinal assessments of individual change.

## 4 Discussion

In this study, an examination of the outcome measures used in RC evaluation studies identified 39 outcome measure and indicator entries across 22 articles. These measures were categorized into a multi-layered framework comprising intra-personal and psychological changes, behavioral and social changes, and system and environment. This breadth indicates that the included evaluations addressed more than symptom reduction, encompassing personal recovery, social participation, and the support environment itself (3, 35).

Particularly, the frequent use of measures assessing intra-personal changes, such as well-being, personal recovery, and empowerment, is consistent with the emphasis of RCs on individuals’ strengths and agency (7). Notably, the repeated use of WEMWBS/SWEMWBS and QPR, in 11 and four articles, respectively, indicates sustained attention to well-being and personal recovery in current evaluation practice. However, frequency of use does not establish these as agreed core outcomes or demonstrate that the instruments are the most meaningful, responsive, or appropriate for RC participants.

Conversely, more than two-thirds of the extracted inventory entries (28 of 39) were used in only a single article, indicating diversity in outcome evaluation. This diversity may reflect the multifaceted and individualized nature of RC practice and legitimate differences in evaluation purposes, populations, and contexts. For example, evaluations involving adults transitioning from homelessness, NHS staff, or participants in creative workshops may reasonably require different outcomes (14, 15, 21). Diversity should therefore not automatically be interpreted as a problematic lack of uniformity. Nevertheless, disparate measures and measurement schedules can hinder comparison when studies address similar questions. Some commonality may support synthesis while retaining outcomes appropriate to particular participants and settings.

Furthermore, this study suggests that important concepts reflecting the unique ethos of RCs may not be adequately represented by the measures used. Although many measures addressed psychological outcomes, direct assessment of learning processes, learner experience, knowledge application, self-directed learning, educational engagement, and transfer into everyday life was comparatively limited. Co-production, learning, belonging, and social inclusion were not entirely absent: studies assessed learning-objective achievement, self-management, individualized goals, social inclusion, loneliness, and recovery-supporting environments (9, 16, 24, 25, 29, 32). However, these related assessments do not necessarily capture the full educational and relational contribution of RCs. This suggests a possible gap between intervention philosophy and evaluation focus. Future evaluations could also consider community-level outcomes, such as reciprocal links with local organizations and opportunities for inclusive participation, where these reflect stakeholder priorities. The question is whether selected measures capture what matters within RC philosophy and practice, rather than simply whether those outcomes are readily measurable.

Existing RC-specific instruments should also be considered. For example, the RECOLLECT Fidelity Measure addresses features including learning, co-production, tailoring provision to the student, and social connectedness (36). Related RECOLLECT Checklists assess alignment with RC principles from student, peer-trainer, and manager perspectives (37). These tools were not used in the included evaluations. Some included studies predated their development, and the timing of availability may have influenced uptake. However, the reasons for their non-use in the included evaluations remain unclear from the available evidence; awareness and alignment with evaluation aims are possible considerations. Their absence therefore does not establish limited use across RC research. The development study was excluded because it evaluated the instrument rather than RC-related outcomes. Whereas many included evaluations focused on individual outcomes, combining fidelity assessment with participant measures could help investigate whether and how adherence to RC principles relates to individual change, learning, and educational outcomes. Researchers and learners should jointly consider existing tools’ coverage, acceptability, and respondent burden when designing such evaluations (38, 39).

Additionally, the use of objective outcomes such as health service utilization data and economic indicators across multiple studies is noteworthy (20, 22, 31). These findings illustrate the value of considering service and institutional outcomes alongside subjective self-report measures. Their interpretation nevertheless requires attention to context: reduced service use alone does not demonstrate improved recovery. Future studies should adopt multifaceted evaluations combining subjective and objective measures where appropriate to their purposes, with educational processes and outcomes considered alongside psychological change.

Regarding psychometric reporting, 15 of the 22 articles cited previous psychometric information or identifiable validation literature, whereas only two explicitly reported reliability estimates calculated from their own data. Six provided neither specific psychometric evidence nor an identifiable validation citation. These findings indicate limitations in reporting, rather than poor measurement quality. Previous validation does not automatically establish suitability for a particular RC population, instrument version, or evaluation purpose. Future reports should distinguish prior evidence from analyses undertaken in the current sample and explain instrument selection. Internal consistency alone does not establish validity or responsiveness and is not applicable to every indicator, including single items and administrative data.

Therefore, future research should prioritize three directions. First, outcomes should be selected according to research purposes and target populations, and the reliability, validity, and responsiveness of existing instruments should be evaluated in the intended RC populations. For self-report scales, the COSMIN Study Design checklist can guide studies of measurement properties, including content validity and the ability to detect change (40). Second, study designs should support the appropriate use of existing measures, with learners, people with lived experience, peer educators, families, educators, clinicians, researchers, and service planners involved in decisions about outcomes, measurement schedules, and feasibility (38, 39). Long-term follow-up could help examine sustained outcomes and the application of learning in everyday life (41). Third, where existing evaluations insufficiently cover outcomes important to RC stakeholders, a co-produced core outcome set could be explored through qualitative work, a Delphi process, and consensus meetings (42). Agreement on what to measure should be distinguished from validation of the instruments used to measure it, and shared outcomes should retain individualized goals and context-specific priorities. Adaptation or development of measures may be considered where important gaps remain. The present inventory can inform these decisions without prescribing a fixed measurement set.

### 4.1 Strengths and limitations

A strength of this review is its systematic mapping of outcome measures and objective indicators, integrating their measured constructs, patterns of use, and reporting of reliability and validity. By making the coverage of existing evaluations more explicit, this mapping can help learners, researchers, and other stakeholders consider whether selected outcomes reflect RC principles and the changes that matter to participants. It provides a basis for co-produced decisions about outcome selection and evaluation design, including how existing measures can address educational, personal, and social priorities and where further assessment of their suitability is needed. In this way, the review can support evaluations that better reflect the educational and co-produced nature of RCs while retaining individualized goals and context-specific priorities.

This study has several limitations. First, this review mapped what researchers have chosen to measure in published quantitative RC evaluations; it did not synthesize intervention effects to determine what RCs actually achieve. Because eligibility required quantitative outcome measures or objective indicators, studies employing exclusively qualitative methods were excluded. Consequently, aspects central to RC philosophy and practice, such as co-production, learning processes, belonging, and relational changes, may not have been fully represented in the extracted inventory. The absence or limited representation of a domain in this review therefore does not demonstrate that RCs have no effect in that domain or that it is unimportant to participants. Studies conducted solely to develop or validate instruments were also excluded. The inventory should thus not be interpreted as a comprehensive catalogue of all available RC measures or all outcomes associated with RC participation.

Second, the diversity of outcome indicators is a substantive finding of this review rather than a limitation in itself. The limitation concerns the context in which patterns of measure use can be interpreted. Included studies differed in their populations, designs, countries, RC operational models, and measurement schedules. Consequently, aggregate frequencies combine measures used for different purposes and under different conditions, limiting direct comparison of measurement choices across studies. The review could describe these patterns but could not determine whether differences in measure use reflected appropriate adaptation to evaluation aims, practical constraints, or inconsistent selection. Frequencies indicate how often an instrument appeared in the included articles, not its importance to participants, responsiveness, or measurement quality. They also count articles rather than necessarily independent study populations. Assigning multi-concept instruments to a single principal domain further simplifies their content and may underrepresent educational or social concepts embedded within measures assigned to other domains.

Third, the synthesis of reliability and validity was based on reporting in the included articles rather than a systematic re-evaluation of the psychometric properties of the instruments themselves. Original validation studies were not independently appraised, and no formal critical appraisal of the included studies was undertaken. The review therefore describes the reporting of psychometric evidence and does not establish the comparative quality of the measures or support definitive recommendations about which instruments should be used.

Fourth, the selected databases and eligibility criteria may have limited coverage. PsycINFO and Scopus were not searched because institutional subscription access was unavailable. Restriction to English-language journal articles also excluded dissertations, non-journal local service reports, and other grey literature. Locally developed indicators and measures used in routine practice or non-English-language settings may therefore be underrepresented. In addition, three reports could not be retrieved. The distribution of measures should be interpreted as describing the included literature rather than the full range of RC evaluation practices.

## 5 Conclusion

This scoping review mapped 39 outcome measure and indicator entries across 22 RC evaluation articles, covering intra-personal and psychological changes, behavioral and social changes, and system and environment. Repeated use of WEMWBS/SWEMWBS and QPR reflected common measurement practices, while the diversity of indicators may reflect differing evaluation purposes and contexts. Direct assessment of educational processes and outcomes was comparatively limited in the included quantitative literature, but this describes what researchers measured rather than what RCs achieved. The mapping can inform co-produced decisions about outcome selection and study design, alongside further evaluation of existing instruments’ reliability, validity, and responsiveness in the intended RC populations. Involving learners and other stakeholders in these decisions can help align evaluation with RC principles and participant priorities while retaining individualized goals and context-specific outcomes.

## Data Availability

The original contributions presented in the study are included in the article/**Supplementary Material**, further inquiries can be directed to the corresponding author/s.
