# Supplementary material for: Outcome indicators used in recovery college effectiveness studies: a scoping review
Source: Front Psychiatry. 2026 Oct 22;17:1969790. doi: 10.3389/fpsyt.2026.1969790 (PMC13736581; doi:10.3389/fpsyt.2026.1969790)
Supplement: Supplementary file 1 [file SupplementaryFile1.docx]

Supplementary Table S1

# Search strategies used for the literature search

Note. PubMed, Web of Science and CINAHL Ultimate were searched on March 8, 2026. ERIC and ProQuest were searched on September 8, 2026. The ProQuest search covered seven databases: Coronavirus Research Database, Education Research Index, MEDLINE, ProQuest Dissertations & Theses A&I, PTSDpubs, Publicly Available Content Database, and Research Library. ERIC and New York Times were not included in the ProQuest search. All searches covered publications dated on or before March 8, 2026, with no lower publication-year limit. Eligibility was restricted during screening to English-language journal articles; conference abstracts, dissertations, newspaper articles and other grey literature were excluded. Strategies were adapted to each search interface. Counts are before removal of duplicate records within and across sources.

## PubMed

Search date: March 8, 2026 | Records retrieved: 80

("Recovery College"[tiab] OR "Recovery Colleges"[tiab] OR "recovery education centre*"[tiab] OR "recovery education center*"[tiab]) AND (outcome*[tiab] OR evaluat*[tiab] OR effect*[tiab] OR impact*[tiab] OR measure*[tiab] OR scale*[tiab] OR questionnaire*[tiab] OR instrument*[tiab])

## Web of Science

Search date: March 8, 2026 | Records retrieved: 40

TS=("Recovery College" OR "Recovery Colleges" OR "recovery education centre*" OR "recovery education center*") AND TS=(outcome* OR evaluat* OR effect* OR impact* OR measure* OR scale* OR questionnaire* OR instrument*)

## CINAHL Ultimate

Search date: March 8, 2026 | Records retrieved: 76

(TI ("Recovery College" OR "Recovery Colleges" OR "recovery education centre*" OR "recovery education center*") OR AB ("Recovery College" OR "Recovery Colleges" OR "recovery education centre*" OR "recovery education center*")) AND (TI (outcome* OR evaluat* OR effect* OR impact* OR measure* OR scale* OR questionnaire* OR instrument*) OR AB (outcome* OR evaluat* OR effect* OR impact* OR measure* OR scale* OR questionnaire* OR instrument*))

## ERIC

Search date: September 8, 2026 | Records retrieved: 13

("Recovery College" OR "Recovery Colleges" OR "recovery education centre" OR "recovery education center")　AND　(outcome* OR evaluat* OR effect* OR impact* OR measure* OR scale* OR questionnaire* OR instrument*)

## ProQuest

Search date: September 8, 2026 | Records retrieved: 127

TI,AB("Recovery College" OR "Recovery Colleges" OR "recovery education centre" OR "recovery education center")　AND　TI,AB(outcome* OR evaluat* OR effect* OR impact* OR measure* OR scale* OR questionnaire* OR instrument*)

Total records retrieved: 336.
