# Supplementary material for: Outcome indicators used in recovery college effectiveness studies: a scoping review
Source: Front Psychiatry. 2026 Oct 22;17:1969790. doi: 10.3389/fpsyt.2026.1969790 (PMC13736581; doi:10.3389/fpsyt.2026.1969790)
Supplement: Supplementary file 2 [file SupplementaryFile2.docx]

Supplementary Table S2

# PRISMA-ScR reporting checklist

**Outcome Indicators Used in Recovery College Effectiveness Studies: A Scoping Review**

Item labels are abbreviated. Locations refer to manuscript sections and the accompanying tables and figure.

| No. | Topic | Location | Reporting detail |
| --- | --- | --- | --- |
| 1 | Title Title identifies a scoping review | Title page | Reported. |
| 2 | Abstract Structured review summary | Abstract | Reported. |
| 3 | Rationale Why this review is needed | Introduction | Distinguishes this measure-level synthesis from earlier RC reviews. |
| 4 | Objectives Review objectives and questions | Introduction | Three review questions specify outcomes, measures and psychometric reporting. |
| 5 | Protocol Protocol availability and registration | Section 2.1 | Protocol prepared, not registered and not publicly available. |
| 6 | Eligibility Selection criteria and rationale | Section 2.3 | Quantitative evaluation focus, publication type, language and exclusions are stated. |
| 7 | Sources Sources and search dates | Section 2.2; Supplementary Table S1 | Initial and additional searches, publication cutoff and seven ProQuest databases are specified. |
| 8 | Search Reproducible search strategy | Supplementary Table S1 | Full strategies for all five search sources are provided. |
| 9 | Selection Screening and eligibility procedures | Section 2.4 | Three independent reviewers; DOI/title deduplication; consensus. |
| 10 | Charting Data charting procedures | Section 2.5 | Joint extraction by YS and AB; seven-article pilot; independent check by MT. |
| 11 | Data items Variables and recording rules | Section 2.5 | Variables, measure consolidation and version-specific recording are described. |
| 12 | Appraisal methods Critical appraisal if undertaken | Section 2.6 | Not applicable: no formal critical appraisal; mapping purpose explained. |
| 13 | Synthesis methods How charted information was synthesized | Section 2.6 | Article-level frequencies, inductive classification and psychometric reporting categories. |
| 14 | Selection results Selection counts and exclusion reasons | Section 3.1; Figure 1 | 336 records; 187 duplicates removed; 149 screened; 122 excluded; 27 sought; 3 not retrieved; 24 assessed; 2 excluded; 22 included. |
| 15 | Characteristics Characteristics and source citations | Section 3.1; Table 1 | Population, design, country, sample size and measurement schedules. |
| 16 | Appraisal results Critical appraisal findings if applicable | Section 2.6 | Not applicable: formal critical appraisal was not undertaken. |
| 17 | Individual results Relevant findings from each source | Tables 1–4; Sections 3.2–3.5 | Study-linked measures, constructs, frequency and psychometric reporting. |
| 18 | Synthesis results Synthesis addressing review questions | Sections 3.2–3.5; Tables 2–4 | 39 entries; three domains; measure-use and reporting summaries. |
| 19 | Evidence summary Interpretation and relevance of findings | Discussion | Educational focus, contextual diversity, existing measures and co-produced evaluation. |
| 20 | Limitations Review limitations | Section 4.1 | Scope, contextual comparability, reporting-based synthesis and retrieval restrictions. |
| 21 | Conclusions Interpretation and implications | Section 5 | Single paragraph; implications for measure selection and study design. |
| 22 | Funding Funding of review and included sources | Funding | Review funding and funder role reported. Funding of included sources is not reported. |

## Reference

Tricco AC, et al. PRISMA Extension for Scoping Reviews (PRISMA-ScR): Checklist and Explanation. Ann Intern Med. 2018;169:467–473. doi:10.7326/M18-0850. Official checklist: https://www.prisma-statement.org/scoping
